# Practical Medicine

**Published:** 1876-10

**Authors:** 


### VI. Practical Medicine.

1. *Disorders of the Vascular System in Burns.* Coustou. (*Jour. de Méd. et de Chir.*, May, 1876.)

These are less common than the attacks of pleurisy, bronchitis, pneumonia and enteritis, so often observed as complications of burns, but are equally interesting. The accidents referred to are phlebitis and the formation of clots in vessels, due to alterations in the fluidity of the blood. Where burns are extensive and accompanied by extensive phlyctenular eruption, an enormous quantity of serosity is withdrawn from the blood, which thus becomes thicker and more readily coagulable. This also accounts for the tendency to syncope in these severe cases. C.'s observations (service of Professor Verneuil) were all of extensive burns of the trunk. In the first case, after five weeks, there was œdema of the left leg, and a hard, resisting cord could be felt along the line of the femoral vessels in the thigh. Phlebitis of the femoral and external iliac veins was discovered, *post mortem.* In a second patient, six weeks after injury, when the wound was progressing to cicatrization and the general health was fair, there occurred phlebitis of the femoral vein and erysipelas, followed by recovery. Eleven days after the burn received by a third patient, there was complete hemiplegia and hemianæsthesia of the entire right side of the trunk, supposed to be due to temporary obliteration of the sylvian artery by a coagulum. In this case the right side of the head and face was simiarly affected.

2. *A Case of Acute Fibrinous Bronchitis with tube casts.* E. D. Worthington, M.D. (*Canada Med. and Surg. Jour.*, June, 1876.)

The patient was a stout English woman, forty-one years old, and the mother of thirteen children, the youngest only three months old. She had for several days, with slight symptoms of fever, "labored respiration." Suddenly she was attacked with severe dyspnœa and violent paroxysms of coughing, with pain in the chest, great distress of countenance, and a livid hue of the skin. She soon succeeded in expectorating a mass of tube casts of the bronchi as large as a tablespoonful. "In a few hours another paroxysm of coughing ended in more expectoration of tube casts, the disappearance of the cyanosis, and most marked relief to the patient." The patient recovered. "Some of the larger specimens had a diameter at the base of fully three-eighths of an inch;" they were cylindrical, white or pearly color, with a few delicately colored pink spots. One mass had twenty-eight terminal branches, and was four and one-half inches long. The patient continued to cough up casts for two months, notwithstanding she recovered her general health, so that at the end of this time she was able to make a journey to England. Dr. W. says this is so rare an affection that "of acute, fibrinous bronchitis, with fibrinous expectoration, Lebert could find but seventeen observations, after a careful analysis of all the cases known at the time of writing." Each cast consisted of a number of laminæ, arranged concentrically, those in the center being folded and involuted. Under an objective of five hundred diameters the laminæ were translucent and structureless; the spaces between them were filled with fine fibrils of fibrine and innumerable leucocytes.

3. *Rare case of Gall Stones discharged through the side.* D. PERLEY, M.D. (*Boston Med. and Surg. Jour.*, June 22, 1876.)

The patient, a male, was seventy-six years old. He had had frequently pain in the region of the liver, and in 1869 an abscess formed in this locality, with great systemic disturbance. The cavity was opened and discharged pus and dark matter; in a month gall stones began to be discharged. The discharge of the stones continued with "volcanic irregularity of rest and activity" till December, 1873, since which time there had been no discharge. The patient's health had greatly improved.

4. *The Menses in Phthisical Patients.* LADMIRAL. (*Lyon Médic.*, No. 22.)

Menstruation and evolution are two distinct and yet closely allied functions; hence the possibility (and this is not unfrequently noted) of conception by a woman whose menstrual flow has disappeared under the influence of a general disease.

Ordinarily the suppression of the menstrual flow, in a woman who coughs and becomes emaciated, is a symptom which points to the rapid evolution of pulmonary tubercolosis. In a large number of patients the catamenial function is suspended at the same time that the disorder of the lungs is advancing; there are rarer cases where the amenorrhœa precedes the habitual manifestations of the diathesis. It is this fact which leads some to look to the uterus for a lesion which does not exist. Hence, *always when there is menstrual suppression without appreciable cause, and when pregnancy cannot be considered possible, physicians should carefully examine the chest and give a reserved diagnosis.* The menstrual flow, however, has no influence upon the march of pulmonary phthisis; but its reappearance always coincides with a period of arrest or cure of the disease (temporary or permanent), and is a proof of the fair condition of the constitution.

Dr. Mollière, in reviewing Ladmiral's treatise, concludes that the author has not been sufficiently explicit on this point, viz.: whether the existence of pregnancy should be admitted or denied when these variations in the menstrual function occur. It is often true that women who are profoundly affected are also apt to conceive; and, *per contra*, certain general symptoms may co-exist with amenorrhœa, and lead to a suspicion of the early stage of a pregnancy which does not exist.

5. *The Abortive Treatment of Whooping Cough by Sulphate of Quinine.* R. K. HINTON, M.D. (*Med. and Surg. Reporter*, Aug. 26, 1876.)

Dr. H. reports ten cases in which this treatment was successful. The ages of some of the patients are omitted in the report, but we gather that the range of their ages was at least from eighteen months to twenty-four years. The doses given were one to five grs. every three to six hours, according to age and severity. In all the cases the pertussis was aborted, the period required varying from a few hours to a week.

Unfortunately, the doctor does not state whether he has had any cases of failure of this treatment, and if he has, how many. The value of his observations are very much lessened by this omission. In order to give children quinine without their tasting or afterward vomiting it, he has the doses rolled up in wafers. A wafer is moistened, to make it soft, and then placed in a spoon nearly full of water; the spoon is reached far back upon the tongue, and its contents emptied into the throat; the wafer is swallowed as readily as the water. "The smallest child can swallow the wafer and retain the quinine without vomiting."

